# The Poor Man's Home.—VII

**Published:** 1897-10-02

**Authors:** 


### Modern Sociology.

### THE POOR MAN'S HOME.—VII.

### "Philanthropy and 4 per cent."

We propose now to speak of some of the most successful semi-philanthropic experiments in housing, this being the basis which is, in our opinion, the soundest for work of the kind. By semi-philanthropic we mean, as has been before explained, work which is philanthropic in intention, but is conducted on sound business principles, and which gives a fair, though not large, return for the money expended. It is, in short, the idea expressed in our heading, "Philanthropy and 4 per cent." It must be understood, however, that 4 per cent. is not an absolute figure, but represents simply the average rate of interest of the locality. It may rise to five or six without reproach to the landlord, or fall to three without implying that the tenant receives a charity. Our title is taken from the Four per Cent. Industrial Dwellings Company in London, which, however, earns more than this, but does not pay dividends above 4 per cent. The remainder, which hitherto has averaged from 2 to 3 per cent., is partly placed to reserve and partly expended in some way for the benefit of tenants. The company owns two large blocks of building in Brady Street containing three and four-room tenements, and two smaller ones containing one-room tenements. The blocks contain also bath-rooms, and houses for the superintendent and the janitor. The blocks are built round an open space of about a third of an acre, part of which is planted with shrubs. There is a club-room and library attached to the buildings. The rent for a single-room tenement is 1s. 6d. a week; for a three-room one, 4s. to 5s. 6d.; and for a four-room flat, 5s. 6d. to 7s. These rents are from 30 to 50 per cent. lower than the rents paid for similar accommodation in the neighbourhood. The Charlotte de Rothschild buildings, owned by the company, provide accommodation for a rather lower class of tenants than the Brady Street houses. Each tenement has a water-closet for itself, and a scullery in which is a boiler. Tenants are forbidden to sub-let their houses, or to take in lodgers. Rents are paid weekly in advance, and tenants on entering must pay a deposit of 5s. if they occupy one of the larger tenements, or 2s. 6d. if they go into one of the single rooms. Each tenement, however small, enters separately from an open stair, so that within its limits each house is self-contained.

This company may be taken as an example of all similar ventures, while in each will be found differences of detail. The Improved Industrial Dwellings Company is probably the largest in London, and owns forty-five different estates in London. It has for many years paid a dividend of 5 per cent., besides building up a large reserve fund. It seems, however, to charge higher rents than the Four Per Cent. Company. The Artisans', Labourers', and General Dwellings Company now owns so many houses meant for the less wealthy of the middle class that it hardly comes within the scope of our inquiry. It began, however, as an association of working men, building houses for their own class. It was originally intended to sell the houses to the occupants on a 99 years' lease. This has been abandoned, because the company could not prevent the occupant selling it if it suited his convenience to leave the locality. He often sold it to some speculator who let it at a higher rental, thus defeating the intentions of the company. Moreover, by thus giving up the ownership the company lost control of the property, and could not prevent overcrowding, or even the establishment of immoral houses. It was only since 1886 that this company had been building blocks for the labouring classes, and, while it provides for a poorer class than many other companies, it economises not by putting poor materials in its buildings, but by such arrangements in building as permit 1,000 persons to the acre to be safely housed. In these blocks the laundry, bath-room, water-closets (two for each floor), sinks, and dust shoots are placed on the landing. While this plan is not so well liked by the tenants as that which gives them all conveniences within their own doors, it is really healthier, and is especially preferable with people so ignorant of sanitation and so careless in their habits as many of the working-class often are. The Metropolitan Association for Improving the Dwellings of the Industrious Classes is the oldest society of the kind in London, and is similar in its methods to those we have spoken of. The Tenement Dwellings Company, however, proceeds on a different plan. It buys up existing property, puts it into good sanitary condition, and lets it. Some of the property of this company is intended for the occupation of the poorest class, and rents are so low as only just to escape causing a deficit. It has, however, houses of a better class, and the rents charged for these, although not higher than the average of the neighbourhood, afford sufficient profit to permit of a dividend of 5 per cent. being paid. One-half of any money earned beyond this goes to a reserve fund, and the other half to a tenants' benefit fund, which is applied as the directors deem wise. In one year a rebate of two weeks' rent was given to each of the tenants. At present the money is devoted to the maintenance of a library at Bolina Road, where the best houses of the company are. These are not blocks, but small houses, each containing two tenements of three rooms and scullery. The rent for each tenement is 6s. 6d. a week.

The Manchester Labourers' Dwellings Company was formed in 1889. Its property was originally a factory in Ancoats, a poor part of the city, which was unused at the time, and consequently was bought cheap. It is to be regretted that the company has not been financially a success. This is due partly to the expense of altering the building for its new purpose being greater than was anticipated, and partly, according to the secretary, because the company cater for "the lowest stratum of workpeople, who, whenever out of work, have not money to pay their rent." Rents are about 6d. a week cheaper in the company's property than they are for similar accommodation in the neighbourhood, and not only are they healthy and convenient, but hot and cold water is run into every kitchen, and gas is provided till eleven o'clock at night. The use of the laundry, however, is charged at the rate of 1d. an hour, and baths, hot or cold, cost 1d. each. In the one and two roomed tenements there are partitioned recesses for beds. In one-room tenements the family must be limited to a husband and wife and two children under five years of age. The rents vary inversely in proportion to height. A single room costs 3s. a week on the fourth storey and 2s. on the seventh. Two-room tenements vary from 4s. 9d. a week in the first three storeys to 3s. 3d. a week on the top floor.

